# Cultural Transmission: Understanding the Processes of Ethnic/Racial Socialization in Racially/Ethnically Minoritized Parents

**DOI:** 10.3390/bs15060716

**Published:** 2025-05-22

**Authors:** Ryan Houston-Dial, Meeta Banerjee, Nada M. Goodrum

**Affiliations:** Department of Psychology, University of South Carolina, Columbia, SC 29208, USA; meeta@mailbox.sc.edu (M.B.); ngoodrum@mailbox.sc.edu (N.M.G.)

**Keywords:** ethnic/racial socialization, ethnic/racial identity, parents, Asian Americans, Black Americans, Hispanic/Latine

## Abstract

Ethnic/racial socialization has been identified as a key protective mechanism within minoritized populations towards racism and discrimination within the United States. Prior research has highlighted the importance of the relation between ethnic/racial identity and ethnic/racial socialization practices, but less is known about how these associations manifest across diverse groups. The current study explored the associations between parental ethnic/racial identity and ethnic/racial socialization in a national sample of 414 Black, Latine, and Asian American parents. Racially/ethnically minoritized parents from around the United States reported on their ethnic/racial identity and ethnic/racial socialization practices with their children. Hierarchical linear regressions indicated that racial centrality and private regard were significant predictors of preparation for bias and cultural socialization messages. The implications from this study are that there are myriad factors that influence socialization practices in racially/ethnically minoritized families.

## 1. Introduction

Within the next 20 years, racial/ethnic minorities are projected to become the majority in the United States. Yet, a growing problem is that the daily experiences of racially/ethnically minoritized youth will continue to be moderated by the macrosystem influences (e.g., policies, programmatic infrastructure) of a White-dominant culture (H. L. Wang, 2021), thus potentially increasing the barriers and biases impacting racially/ethnically minoritized individuals. Thus, racially/ethnically minoritized individuals have crafted adaptive cultures to navigate inequitable systems (García Coll et al., 1996). One such adaptive cultural mechanism is the process of ethnic/racial socialization which encompasses giving racially/ethnically minoritized youth messages about race, ethnicity, and culture. Ethnic/racial socialization (ERS) is a multidimensional concept defined as the transmission of attitudes, beliefs, and messages about group membership (Hughes et al., 2006). There is a need to understand how racially/ethnically minoritized parents are currently preparing their children to navigate systems of bias and discrimination. It is imperative to study the importance of cultural values due to their ability to provide effective coping strategies for overcoming systems of injustice. Research has suggested there is a strong association between ethnic/racial identity (ERI) and the likelihood of ERS messages being passed to children (Hughes et al., 2006; Kiang et al., 2023). Moreover, parents who held higher positive associations with their ERI felt more equipped to pass down ERS messaging to their children (Kiang et al., 2023).

Although research has shown us that ERS practices are important in minoritized populations (M.-T. Wang et al., 2020a, 2020b; Huguley et al., 2019; Stevenson, 1995), studies have not looked at these processes in a national multiethnic/multiracial sample across the United States. The current study examined how parental ethnic/racial identity is associated with ethnic/racial socialization practices within a diverse sample of parents.

### 1.1. Theoretical Frameworks

Vital parenting practices such as ethnic/racial socialization in racially/ethnically minoritized families have been well documented, indicating that key theoretical frameworks help to pinpoint the process. One such framework is García Coll et al.’s (1996) Integrative Model for the Study of Developmental Competencies in Minority Children, which highlights how parenting practices such as ethnic/racial socialization are associated with youth development across minoritized populations. The integrative model posits that the effect of social position variables, in particular race/ethnicity, may be associated with adaptive cultural mechanisms, such as ethnic/racial socialization (ERS). The burgeoning field of research indicates that the practice of ERS is a protective factor for racially/ethnically minoritized youth (M.-T. Wang et al., 2020a, 2020b). Specifically, adaptive culture assists with shaping family roles and structure for ethnically/racially minoritized youth, and familial values and beliefs therefore allow parents to socialize their children around race, ethnicity, and culture.

Another framework that illustrates how racially/ethnically minoritized families navigate identity is Sellers et al.’s (1998) Multidimensional Model of Racial Identity (MMRI). Sellers et al. (1998) described racial identity in Black individuals as the qualitative meaning that ethnic group membership is incorporated to one’s self concept. The component of racial centrality is based on the degree an individual’s racial identity is a prominent part of their self-concept. Furthermore, regard refers to the affective and evaluative judgments that are held about one’s own ethnic racial group membership and can include both private and public regard (Sellers et al., 1998). Private regard is the extent to which individuals feel positively or negatively about their ethnic group and membership. Public regard refers to how others in society view a particular ethnic/racial group. The current study focused on private regard and centrality. Overall, through the multidimensionality of ethnic/racial identity development, there is a unique opportunity to explore whether and how identity association can be a catalyst towards ERS practices from parent to child.

### 1.2. ERI Development in Children of Color

The development of racially/ethnically minoritized children needs to consider how contextual experiences influence identity development. Ethnic/racial identity (ERI) is a unique multidimensional construct that takes into account the feelings and attitudes of group membership over time for ethnic minority individuals (Umaña-Taylor et al., 2014). The contributions of research for ERI through the use of Sellers et al. (1998) amongst others has allowed scholars to examine ERI construction in minoritized groups. Research has shown support for the effectiveness of ERS messages influencing ERI development in children of color with positive psychosocial outcomes (Hughes et al., 2006; Stevenson & Arrington, 2009; Neblett et al., 2013; Rivas-Drake, 2011; Rivas-Drake et al., 2014; Witherspoon et al., 2021). More specifically, Stevenson and Arrington (2009) were able to locate findings that ERS messaging mediated the relationship between racism exposure and ethnic/racial identity dimensions such as centrality (Stevenson & Arrington, 2009). Furthermore, youth who reported an awareness of discrimination through ERS messaging still held favorable attitudes towards being African American. Similarly, in Latine and Asian American children evidence was found for ERS endorsement producing positive outcomes towards identity development. For example, Witherspoon et al. (2021) examined the influence of neighborhood contextual influences and acts of discrimination on ERI development. More specifically ERS messaging was positively associated with Latine youth’s ERI components of private regard and centrality. This result suggests that Latine youth who receive messages centered around ethnic pride believed that their identity formation is a central component in their self-image. As a result, this also led to more positive attitudes towards their ethnic minoritized group (Witherspoon et al., 2021). Asian American youth also reported a relationship between receiving ERS messaging and ERI construction (Kiang et al., 2019; Woo et al., 2020; Yip, 2008). Asian American youth who received ERS messages reported an increase in belongingness to an ethnic minority group as well as a stronger association with their ERI (Kiang et al., 2019).

It is beneficial to understand how ERI develops as ethnic minority children receive socialization messages from their parents about group membership and society. Understanding the linkage between ERS and ERI prompts the protective factors that ERS messages provide for ethnic pride and resilience in ethnic minoritized children. Additionally, as parents continue to supply their children with socialization messages, this influences identity development. Furthermore, through this process, parents are able to provide mechanisms to preserve their culture. Studying the connection, in particular, sheds light on how these messages have changed and have been passed down through generations, empowering ethnic minority children with the skills they need to succeed in a society dominated by White culture. This study will examine more closely how a parent’s ERI construction influences the likelihood that certain ERS messaging will be passed on to their children.

### 1.3. Ethnic/Racial Socialization as a Construct

Ethnic/racial socialization (ERS) has been categorized into four dimensions: cultural socialization, preparation for bias, egalitarianism, and promotion of mistrust (Hughes & Chen, 1999; Hughes et al., 2006). The current study focused on the exploring the dimensions of cultural socialization and preparation for bias in racially/ethnically minoritized populations. Cultural socialization can be defined as emphasizing ethnic heritage through participating in practices and messages that expose their children to the traditions of their racial/ethnic group (e.g., celebration of holidays, food, clothing; Hughes et al., 2006). Additionally, preparation for bias encompasses discussing racial bias and discrimination that youth may experience (M.-T. Wang et al., 2020a, 2020b). Parents may model appropriate behaviors when encountering discrimination, build awareness, and provide coping mechanisms. These practices have been linked with better academic and mental health outcomes in the face of environmental/social stressors for racially/ethnically minoritized children who received cultural socialization and preparation-for-bias messages (Ayón et al., 2020; Kiang et al., 2023; Umaña-Taylor & Hill, 2020; M.-T. Wang et al., 2020a, 2020b). The existing literature has shown that ERS practices are adaptive across racially/ethnically minoritized families (M.-T. Wang et al., 2020a, 2020b); however, few, if any, studies have focused on examining ERS in Black American, Asian American, and Latine families simultaneously.

### 1.4. Highlighting the Importance of ERS in Black Families

Throughout changing sociopolitical contexts, ERS dimensions such as cultural socialization and preparation for bias has served as a protective factor against bias and discrimination for Black children in the United States (H. L. Wang, 2021). The conceptualization of ERS can be attributed to Black researchers who investigated ERS practices in Black families, which facilitated a better understanding of Latine and Asian American families (Bowman & Howard, 1985; Demo & Hughes, 1990). In their landmark study, Bowman and Howard (1985) indicated that Black youth who did not receive ERS messages reported lower self-efficacy than children who did, highlighting the importance of ERS within the academic trajectories of Black youth.

Additionally, the existing literature has shown that ERS practices and messaging has led to gains in both the psychosocial realm (e.g., emotion regulation, less depressive symptoms) and academic spheres for Black youth (Evans et al., 2012; Dunbar et al., 2015). Black youth that are socialized around Black cultural knowledge and traditions also report positive attitudes towards being Black (Demo & Hughes, 1990). Youth who receive more cultural socialization messages are more often engaged in school, and thus more likely to perform better (Banerjee et al., 2017; Umaña-Taylor & Hill, 2020; M.-T. Wang et al., 2020b). These results speak to coping strategies that a sense of ethnic pride provide for Black children in the face of societal demands. Similarly, there is evidence that Black parents were more likely than other racially/ethnically minoritized group parents to use preparation-for-bias messages (Hughes et al., 2009). Neblett et al. (2009) found that Black boys were more persistent in difficult academic tasks than Black boys who did not. Thus, preparation-for-bias messages is a key component in Black youth coping with racism and discrimination in multiple contexts.

### 1.5. Exploring ERS Practices in Latine and Asian Populations

Although Latine and Asian populations have been less studied, the benefits of providing ERS to youth have been observed (Kulish et al., 2019; Young et al., 2021). Cultural socialization practices in Latine populations are composed of specific messaging that is relevant to their cultural heritage (Nieri et al., 2022). Through cultural socialization, youth reported a stronger ethnic/racial identity (Rivas-Drake, 2011; Rivas-Drake & Marchand, 2016). Additionally, Latine parents were more likely to bring up immigration status than other racially/ethnically minoritized parents regarding preparation for bias in discussing prejudice and discrimination in the U.S. (Ayón et al., 2019; Spears-Brown et al., 2022; Eyal et al., 2022). Eyal et al. (2022) highlighted specific methods Latine parents used to comfort their child in the face of discrimination in that parents modeled appropriate responses towards discrimination and their usage of preparation-of-bias messages was associated with strengthening their children’s self-esteem. Moreover, Latine youth were able to recognize discrimination better at school when families discussed potential acts of discrimination (Spears-Brown et al., 2022).

While research suggests that Asian American parents are less likely than Black and Latine parents to engage in ERS, messaging still occurs within families (Hughes et al., 2008; Rivas-Drake et al., 2009). Similarly to Black and Latine populations, cultural socialization was associated with a stronger identification with ethnic/racial group membership in Asian American populations (Tran & Lee, 2010; Raval et al., 2025). For example, Daga and Raval (2018), found that South Asian parents were more likely to endorse cultural socialization messages compared to preparation-for-bias messages. Similar results were found in a qualitative study conducted with South Asian youth and parents, where parents reported being much more comfortable with providing cultural socialization messages rather than preparation-for-bias messages to their children (Patel et al., 2023). Furthermore, Huynh and Fuligni (2008) showed that a sample of Chinese American parents endorsed more cultural socialization and preparation-for-bias messages to their children comparative to their White counterparts. Additionally, Asian American youth who received preparation-for-bias messages were more likely to perform better in school (Seol et al., 2016). Asian American parents were found to engage in practices that reflected preparation for bias (Juang et al., 2016). More research is needed regarding the preparation-for-bias messages Asian American parents are using with their children. Due to the COVID-19 pandemic, racial discrimination has become alarmingly prevalent towards Asian American populations (Huynh et al., 2022), but it is not clear if there was an increase in preparation-for-bias messaging from Asian American families.

Overall, cultural socialization and preparation-for-bias messages have been found to be protective of racially/ethnically minoritized youth development. Yet, research has been limited in examining the context in which racially/ethnically minoritized parents pass down meaningful messages to their children about cultural socialization and preparation for bias. Therein lies a gap to better understand factors affect the likelihood of ERS messages in racially/ethnically minoritized families such as a parent’s ethnic/racial identity association.

### 1.6. Current Study

Like Black populations, there has been a call to study ethnic/racial socialization across other racially/ethnically minoritized groups in the United States, such as Latine and Asian American families (Rivas-Drake et al., 2009; Tran & Lee, 2010; Young et al., 2021; Spears-Brown et al., 2022). However, rarely have these three racially/ethnically minoritized groups been studied together in terms of these phenomena. The evidence indicates that parents are a vital facilitator of ERS messages for racially/ethnically minoritized youth (M.-T. Wang et al., 2020a). Parents are actively equipping their children with tools that help solidify their own association with an ethnic/racial identity (ERI) and ethnic pride. This study aims to understand the content transmission of ERS messages, in particular, cultural socialization and preparation for bias for a diverse sample. Given the prior research showing variability in ERS practices across groups, we hypothesized that there would be group differences in the content of ERS messages between racially/ethnically minoritized parents and differences in the frequency of ERS messages. Furthermore, it is important to understand a parent’s association with their ERI, specifically the relation between ERI and the endorsement of cultural socialization and preparation for bias. We hypothesized that parents with a stronger ERI association (i.e., centrality and private regard) would be more likely to employ ERS strategies (i.e., cultural socialization and preparation of bias) with their child.

## 2. Methods

### 2.1. Procedure

Participants were recruited from a national survey conducted in 2021 through Amazon Mechanical Turk to understand parenting practices across individuals who identified as African American, Asian American, and Latine. Amazon Mechanical Turk is a crowdsourcing platform that allows study researchers to connect with a wide range of individuals around the United States to complete surveys. The study was approved by the Institutional Review Board at the University of South Carolina. Participants were recruited through a flyer on the Amazon Mechanical Turk platform that sought individuals who identified as ethnically/racially minoritized individuals who had children between the ages of 0–18. To ensure confidentiality, surveyors were assigned an MTURK ID to remain anonymous. Based on goals for the study, participants who did not identify as Black, Asian American, or Latine were excluded from the study. Additional eligibility criteria were that participants had to be at least age 18 and have at least one child. Participants were compensated for their time through Amazon MTURK upon completion of the survey.

### 2.2. Sample

The sample for this study comprised 414 Black, Asian American, and Latine parents across the United States. African Americans (*N* = 223) were the largest number followed by Latine (*N* = 122) and Asian Americans (*N* = 69). On average, participants reported the median age of their children was approximately 7 years of age (*SD* = 4.37); aged 3–17. Sixty-nine percent of participants identified as male. Regarding participant age, the majority (55.3%) of the sample comprised parents aged 26–34. The sample was fairly educated in that on average participants held a bachelor’s degree, and most participants were married and living together with their partner (82%) followed by those who were single (18%).

### 2.3. Measures

#### 2.3.1. Ethnic/Racial Identity (ERI)

The Multidimensional Inventory for Black Identity-Short (MIBI-S) scale (Sellers et al., 1998) was used to measure perceptions of their own ERI. Participants indicated their level of agreement on a 5-point Likert scale ranging from “Strongly agree” (1) to “Strongly disagree” (5). The shortened scale consisted of eleven items that assessed two dimensions of ERI: private regard and racial centrality. *Racial centrality* (α = 0.74) is a subscale that measured how much a participant’s self-concept is influenced by their racial group membership, and the 8-item subscale included items such as “Overall, my ethnicity has very little to do with how I feel about myself” and “My ethnicity is important to me”. The *private regard* subscale (α = 0.85) measured one’s attitudes and self-perception towards belonging to their own race or ethnic group. The private regard subscale is composed of 3 items, with example items of private regard being “I am happy that I am my ethnicity” and “I feel good about people of my ethnicity”.

#### 2.3.2. Current Ethnic Racial Socialization Practices

Hughes and Chen’s (1999) ethnic racial socialization measure was used to assess the number of times participants talked to their child about race and ethnicity. The ethnic racial socialization scale was based on a 9-item scale, with subscales for two dimensions of ERS: *cultural socialization* and *preparation for bias*. Parents were asked to recall within the past year they provided ERS messages to their child on a five-point Likert scale ranging from “Never” (1) to “6 or more times” (5). The cultural socialization subscale (α = 0.90) measured participants’ messages imparted to their child concerning cultural pride or traditions. The subscale included 4 items, an example item being “it is important to follow the traditions of your racial or ethnic group”. The preparation-for-bias subscale (α = 0.93) measured participants’ messages imparted to their child concerning potential bias and discrimination while providing coping mechanisms. The subscale included 5 items, with an example item being “you may have hard times being accepted in this society because of your race or ethnicity”.

### 2.4. Data Analysis Plan

A preliminary analysis of the means, standard deviation, and correlations were conducted to examine the relationships between ethnic racial identity association and current ERS practices. To examine whether ERS practices such as cultural socialization and preparation for bias differed between racially/ethnically minoritized groups, a multivariate analysis of covariance (MANCOVA) was run. Participant ethnicity served as a fixed factor, while controlling for the participant child’s age and gender within the model to measure the frequency of ERS practices. Furthermore, hierarchical regression models were developed for both endorsement of cultural socialization and preparation-for-bias messages to understand the association between ERI and decisions on ERS endorsement. Within the models, the variables of participant ethnicity as well as the child’s age and gender were controlled in the first step. In the second step, the ERI dimensions of racial centrality and private regard were the independent variables. Cultural socialization and preparation for bias were the dependent variables for the regressions.

## 3. Results

*Preliminary Analyses.* Descriptive analyses of the means, standard deviations, and correlations between the variables were conducted (Table 1). On average, participants reported providing preparation-for-bias messages and cultural socialization practices at a moderate level to their own children. There was a significant positive relationship between racial centrality and private regard with current ERS practices. Participants who reported higher levels of racial centrality and private regard also reported providing greater preparation-of-bias and cultural socialization messages.

### 3.1. MANCOVAS

A one-way MANCOVA was conducted to determine whether the endorsement of current ERS practices differed across racially/ethnically minoritized groups while covarying child age and gender. There was no evidence of support of the first hypothesis of the study. The overall model was found to be significant (F [2,388] = 4.46, *p* = 0.001; Λ = 0.96). There was not a significant effect of participant ethnicity on the endorsement of cultural socialization or preparation-of-bias messages (Table 2). Therefore, Black/African American, Asian American, and Latine parents reported similar levels of cultural socialization and preparation-for-bias messages (Table 2).

### 3.2. Hierarchal Regression of Study Variables

Two hierarchical regression models were conducted to test if ethnic identity association (i.e., centrality and public regard) significantly predicted current ERS practices (i.e., cultural socialization and preparation of bias) (Table 3 and Table 4). Participant ethnicity, child gender, and child age were included as control variables in all regression models. There was support for the second hypothesis of this study relative to ERI associations affecting the frequency of ERS messages from parent to child. On one hierarchal regression model the results indicated the two predictors (i.e., centrality and private regard) explained 10.2% of the variance (R^2^ = 0.10, F (6,391) = 7.28, *p* < 0.01) for cultural socialization. Furthermore, it was found that centrality negatively predicted cultural socialization messaging (β = −0.18, *p* = 0.002), and positively predicted private regard (β = 0.28, *p* < 0.001).

The results indicated the two predictors (i.e., centrality and private regard) explained 15.5% of the variance (R^2^ = 0.15, F (6,387) = 11.63, *p* < 0.001) for preparation for bias. Moreover, centrality negatively predicted preparation-for-bias messaging (β = −0.11, *p* = 0.05) and positively predicted private regard (β = 0.38, *p* < 0.001).

## 4. Discussion

The current study aimed to understand linkages between parental ethnic/racial identity and their endorsement of ERS messages amongst a multiethnic and multiracial sample in the United States. Surprisingly, there was no evidence of differences in ERS practices among three populations, whereas there was evidence showing that parental ERI played a critical role in the endorsement of ERS messages among Black, Latine, and Asian American families. Research examining ERS practices has often examined racially/ethnically minoritized groups independently rather than altogether in a diverse sample of parents. The current study provides an increased understanding of messaging centered around cultural socialization and preparation for bias in a diverse sample of racially/ethnically minoritized parents. The results contribute to the current understanding of communication between racially/ethnically minoritized parents and children about the salient nature of race within the existing research. Moreover, it showcased the integral factor that ERI may play in the relationship of parents endorsing ERS messages to their children.

Furthermore, this study adds a unique perspective to existing research in that most of the sample were fathers. While gender was not a direct focus of the study, typically, the research on socialization practices has comprised samples that were more representative of mothers. Through this lens of parenting practices, this work supports previous research by Cooper et al. (2019), which highlighted the importance of fathers in relation to ERS practices. As mentioned by García Coll et al. (1996), daily lived experiences for people of color are often shaped through intersectional/social position variables (e.g., gender, ethnicity, etc.) which constructs a unique perspective for a father to share with their children. Exploring a sample that consists primarily of fathers opens the door for nuanced conceptualization of ERS practices between fathers and their children. Further, fathers’ methods of race-related socialization may complement or differ from a mother’s approach than previously theorized. Therein lies an opportunity to inquire about ERS strategies from fathers and their approach towards building positive development of their child’s ERI.

Moreover, while participant age was also not a direct focus of the study, the results representing perspectives from younger parents (e.g., 26–34) is noteworthy. Younger parents transition into later adulthood with a stronger sense of identity and values that influence parenting practices. Navigating modern cultural dynamics and discrimination likely shapes ERS messaging to be nuanced within contemporary issues. These parents offer important insights on changing patterns of ERS messaging since they are situated between younger children forming their own identities and older generations with distinct socialization techniques. Their perspectives are essential for understanding how ERS evolves across generations.

### 4.1. Deconstructing ERS Practices Among a Multiethnic/Multiracial Sample

The first objective of the study was to understand the differences in the endorsement of cultural socialization and preparation-for-bias messages amongst a diverse, multiracial/multiethnic national sample of families. Interestingly, the results indicated that there was not a significant difference in preparation-for-bias messaging or cultural socialization practices among the three populations. Although there was not a significant difference across groups, the means for each group suggested participants were endorsing these messages at a frequent rate (e.g., 3–5 times in the past year) towards their children, suggesting that parents from diverse backgrounds engage in both dimensions of ERS. Our findings suggest that regardless of minoritized parents’ racial/ethnic background, they may value the potential protective nature of endorsing these messages to their children. As mentioned before, the majority of participants in our sample were fathers, indicating that fathers felt that both types of messaging were imperative to provide to their children, especially as they are on the cusp of adolescence.

Moreover, another interesting note is that the data may reflect a general increase in awareness about racism and discrimination in that data collection occurred approximately a year after the racial reckoning that occurred around the death of George Floyd. There is a possibility that through these social events, parents felt compelled to have crucial conversations with their children. Kiang et al. (2022) found support for the outcome that the awareness of discrimination during COVID-19 produced greater identity exploration and fewer socialization messages that minimized race. Furthermore, the awareness of discrimination against other minoritized groups was associated with increased post COVID-19 activism (Kiang et al., 2022). Seemingly, parents considered this process vital in order to communicate to their children the racial injustice that exists within the US. Through conversations with their children, parents can emphasize the importance of cultural pride and equity in society.

### 4.2. Influence of Parental ERI on ERS Practices

The second hypothesis of the study aimed to explore if dimensions of ERI (i.e., centrality and private regard) predicted outcomes such as cultural socialization and preparation-of-bias messaging. The findings confirmed that the specific ERI dimensions of centrality and private regard were significant predictors towards messages centered around ethnic pride and discrimination within the US. The environmental context that shapes the daily experiences between racially/ethnically minoritized individuals may differ in system level influences (Bronfenbrenner, 1979). In particular, components of an individual’s microsystem (e.g., families, school, peers) may be interacting with larger macrosystem components (e.g., policies, programmatic infrastructure). If individuals encounter unfair treatment on the basis of race or witnessing current events that expose unfair practices in the workplace are encoded as discriminatory, it may signal the saliency of their ethnic/racial identity and bring it to the forefront.

These results suggest individuals who reported higher associations with private regard, meaning their own feelings about their racial/ethnic ingroup, were more likely to engage in the process of preparation-of-bias and cultural socialization messages. In comparison, individuals who reported higher levels of centrality were less likely to engage in messages of cultural socialization or preparation for bias. This finding may imply that rather than endorsing particular ERS messages, those with higher racial centrality may feel it is vital to model the importance of their cultural background through everyday activities with children (e.g., language, cultural values, etc.). Racial ideologies which encompass specific dimensions of assimilation (e.g., similarities between African Americans and other nationalities) or nationalism (e.g., the uniqueness of being from African descent) were not a focus of this study but are a key component of the MMRI (Sellers et al., 1998). It is imperative to mention that those dimensions shed light to how nuanced identity development may be for individuals as well as how it may impact their endorsement of certain messages. Individuals who lean towards a nationalistic view may maintain the importance of their own racial ingroup and endorse higher racial centrality, whereas others who hold stronger assimilation beliefs may be more likely to believe other individuals hold them in high regard (e.g., public regard).

Furthermore, it could be that where the individual resides, as well as the demographic composition of the neighborhoods, such as racial diversity, may be influencing the type of messaging they provide their children. Research by Doucet et al. (2018) suggested that the diversity of the neighborhood played a role in the types of ERS messaging parents decided to endorse with their preschool children. In this study, it could be that neighborhood diversity is influencing how parental ERI could be functioning in their endorsement of ERS practices.

### 4.3. Limitations and Directions for Future Research

The present study has many strengths that add to the current body of ERS literature, such as the diversity of the sample from across the United States. However, despite these strengths, the present limitations should be addressed. First, there were no reported outcomes for the children, such as social-emotional well-being as it relates to ERS messaging, which is crucial in illustrating the benefits ERS messaging may provide for racially/ethnically minoritized youth. Future studies should observe youth well-being in relation to received ERS messages in a similar fashion in that explores youth across the country. Furthermore, more in-depth examinations are needed regarding the regional location effect on the frequency of messages imparted to racially/ethnically minoritized youth from parents, including factors such as the prevalence of prejudice and discrimination, the socioeconomic status of an area, and community cohesion. This may allow researchers to understand the socio-demographic factors that prompts the emergence of ERS usage from parent to child, and, moreover, the process by which these practices are passed down in families via intergenerational transmission. This may help to deepen our understanding of the impact of who may be imparting ERS messages to racially/ethnically minoritized youth. Intergenerational transmission also helps explain the preservation of cultural values and knowledge that future generations can utilize for resilience and functional well-being.

### 4.4. Implications and Conclusion

There are several implications of this study, which examined ERS practices among a diverse sample of Black, Latine, and Asian American parents. The psychosocial trajectories of racially/ethnically minoritized children are influenced by the macrosystem-level context in which they reside (Bronfenbrenner, 1979). Studying the varying levels of ERS message frequency among a diverse sample has been minimally explored before. Moreover, this study adds to the conceptualization of ERI’s involvement in facilitating ERS messages from parent to child in research. ERI has been examined as a catalyst of transmission and remains a vital process to continue to understand. This is due to the fact that ERI may help us to develop a deeper understanding of the factors associated with the passage of ethnic pride and prevalence of discrimination in the US. When children of color receive messaging that affirms their cultural background, it cultivates a positive self-concept and a sense of belonging. This understanding has implications in differing settings (e.g., schools, homes, etc.) that allows parents to create supportive environments and facilitate empowerment towards children’s identities.

This study underscores the important point that ethnic/racial socialization is a protective factor for racially/ethnically minoritized children navigating systems of racism and discrimination. This study provides an understanding of factors influencing the frequency of ERS messages from parent to child such as ethnic/racial identity. The implications from this study include similarities in socialization practices between racially/ethnically minoritized groups and a parent’s ERI as an influence on message transmission. There is a need to continue to study ERS practices in racially/ethnically minoritized families. While society is growing to become more inclusive, the impact of discrimination persists, and it is imperative to understand the resources racially/ethnically minoritized youth have, in an effort to be resilient.

## Figures and Tables

**Table 1:** Means, standard deviations, and correlations of study variables.

	1	2	3	4	M (SD)
1. Preparation for Bias	—				2.89
					(1.03)
2. Cultural Socialization	0.79 **	—			2.96
					(0.94)
3. MIBI-Centrality	0.11 *	−0.01	—		2.73
					(0.56)
4. MIBI-Private Regard	0.34 **	0.21 **	0.56 **	—	2.72
					(1.09)

Note: * *p* < 0.05; ** *p* < 0.01; PFB = prep for bias; CS = cultural socialization.

**Table 2:** One way-MANCOVA table on racially/ethnically minoritized groups by ERS strategies.

Dimension of ERS	Black		Latine		Asian American		*F* (2,387)
	*M*	*SE*	*M*	*SE*	*M*	*SE*	
Cultural Socialization	2.90	0.88	3.01	0.90	2.90	1.14	0.716
Preparation for Bias	2.92	0.94	2.96	0.97	2.56	1.27	2.90

Note: SE = standard error.

**Table 3:** Hierarchical regression examining ERI association on ERS practices.

Cultural Socialization	*B*	*SE B*	β	R^2^	ΔR^2^
**Step 1**				0.04	
Black Parents	−0.07	0.13	−0.03		
Latine Parents	0.06	0.14	0.03		
Child Age	0.03	0.01	0.15		
Child Gender	−0.30	0.10	−0.14		
**Step 2**					
MIBI-Centrality	0.31 *	0.09	−0.18	0.10	0.07
MIBI-Private Regard	0.24 *	0.05	0.28		

Note: The variables “Black Parents” and “Latine Parents” were dummy variables used to include ethnicity groups. * *p* < 0.01.

**Table 4:** Hierarchical regression examining ERI association on ERS practices.

Preparation for Bias	*B*	*SE B*	β	R^2^	ΔR^2^
**Step 1**				0.04	
Black Parents	0.28	0.14	0.14		
Latine Parents	0.36	0.15	0.16		
Child Age	0.02	0.01	0.08		
Child Gender	−0.33	0.11	−0.14		
**Step 2**				0.15	0.11
MIBI-Centrality	−0.20 *	0.10	−0.11		
MIBI-Private Regard	0.36 **	0.05	0.38		

Note: The variables “Black Parents” and “Latine Parents” were dummy variables used to include ethnicity groups. * *p* < 0.05. ** *p* < 0.01.

## Data Availability

The original contributions presented in this study are included in the article. Further inquiries can be directed to the corresponding author.
